# Neural correlates and plasticity of explicit emotion regulation following the experience of trauma

**DOI:** 10.3389/fnbeh.2025.1523035

**Published:** 2025-02-13

**Authors:** Annika C. Konrad, Andrei C. Miu, Sebastian Trautmann, Philipp Kanske

**Affiliations:** ^1^Clinical Psychology and Behavioral Neuroscience, Institute of Clinical Psychology and Psychotherapy, Technische Universität Dresden, Dresden, Germany; ^2^Department of Psychology, Faculty of Psychology and Educational Sciences, Babeș-Bolyai University, Cluj-Napoca, Romania; ^3^Insitute for Clinical Psychology and Psychotherapy, Medical School Hamburg, Hamburg, Germany

**Keywords:** PTSD, trauma, emotion regulation, reappraisal, compassion, suppression, fMRI, neural plasticity

## Abstract

Experiencing trauma or other adverse life events is highly prevalent and poses a significant risk for the development of mental disorders. Understanding the underlying mechanisms and neural processes involved in trauma processing is crucial for both prevention and targeting symptoms. Especially, difficulties in emotion regulation emerge as one key mechanism implicated in the development of conditions such as post-traumatic stress disorder (PTSD) following traumatic experiences. However, neural correlates of explicit emotion regulation among individuals who have undergone trauma have not received much attention. Understanding the neural basis of dysregulated emotion following trauma could reveal important details about how trauma interferes with emotional regulation systems, informing the development of more specific intervention approaches. Therefore, this mini review summarizes current research, and identifies relevant gaps in the literature and challenges for future studies. Specifically, it provides an overview of the neural dysregulation associated with explicit emotion regulation strategies such as reappraisal or suppression. Finally, it highlights promising findings from intervention studies targeting emotion regulation, such as trauma-focused exposure therapy and neurofeedback, indicating neural plasticity in individuals with traumatic experiences. Hereby, this review aims to bridge the gap between fundamental and intervention research and highlights future directions for translational research.

## 1 Introduction

The experience of a traumatic event is not only deeply impactful in itself but is often followed by a range of mental health symptoms. However, only a minority of trauma-exposed individuals develop a full-blown mental disorder in the aftermath of a traumatic event (e.g., Koenen et al., 2017). In order to identify individuals at risk, it seems crucial to investigate specific mechanisms for the development of psychopathology. In particular, difficulties in emotion regulation have been proposed as a transdiagnostic mechanism that plays a central role in various mental disorders, including post-traumatic stress disorder (PTSD; Ehlers and Clark, 2000; Fitzgerald et al., 2018).

Emotion regulation has been defined as the conscious or unconscious process of modifying the intensity or type of emotions (Gross, 1998). Given this definition, it is not surprising that individuals who have experienced a trauma and also show difficulties in managing negative emotions appear to be more vulnerable to developing psychopathology (McLaughlin and Lambert, 2017; McLaughlin et al., 2020). While explicit regulation involves a deliberate effort to initiate and monitor the implementation process, implicit regulation describes rather an automatic process happening often without insight (Gyurak et al., 2011). Thus, explicit emotion regulation can be more easily articulated and consciously addressed, making it an important target for therapeutic interventions and a critical focus for psychotherapy research (e.g., Ehlers and Clark, 2000). In general, explicit emotion regulation encompasses many different strategies usually measured by self-report or by specific tasks in which the experimenter demands participants to apply the specific strategy in comparison to a control condition (e.g., passive viewing). Strategies, such as avoidance, suppression, and rumination have been positively, and problem solving and reappraisal negatively associated with psychopathology (Aldao et al., 2010).

With regard to the experience of trauma, a number of studies using self-report measures have indeed provided evidence that explicit emotion regulation strategies, such as rumination, suppression, and reappraisal, serve as mediators between childhood adversity and general psychopathology (for meta-analysis, see Miu et al., 2022). Additionally, other meta-analyses have shown positive associations between rumination or suppression and specifically PTSD symptoms (Seligowski et al., 2015; Miethe et al., 2023), but not for reappraisal (Seligowski et al., 2015).

Although the use of self-report measures is undeniably valuable to assess changes in explicit emotion regulation after trauma exposure and how it contributes to psychopathology, they cannot capture underlying processes that are common to or distinguish between different strategies. Here, the use of neuroscientific methods shows great promise to explore such common or distinct underlying mechanisms. Highlighting differences between explicit emotion regulation strategies following trauma exposure may provide a more comprehensive understanding of emotion regulation difficulties as a transdiagnostic mechanism following trauma, which in turn may inform the development of interventions. To our knowledge, three neuroscientific reviews have included studies of explicit emotion regulation in the context of trauma or PTSD. While Fitzgerald et al. (2018) and Zilverstand et al. (2017) only reviewed two studies, Norbury et al. (2023) solely focused on reappraisal, disregarding other strategies. Conversely, neural pathways involved in automatic forms of emotion processing related to trauma, including passive viewing of emotional stimuli or implicit emotion regulation, have received more attention (for meta-analysis or review, see Hayes et al., 2012; Fitzgerald et al., 2018). This highlights the lack of a comprehensive review synthesizing the current state of the literature on neural correlates of explicit emotion regulation following trauma.

Therefore, we aim to first summarize studies reporting neural correlates of explicit emotion regulation strategies (in response to negative stimuli) in trauma-exposed samples. By including trauma-exposed individuals with and without PTSD, we aim to explore general effects of trauma exposure, while between-group differences may pinpoint alterations in emotion regulation as a specific correlate of PTSD symptoms. Second, we highlight research gaps, and third, we discuss current and future developments in the field of intervention research investigating the neural plasticity of emotion regulation. Being able to show neural plasticity of explicit emotion regulation offers a further level of evaluating the long-term effectiveness of these interventions and their underlying processes.

## 2 Neural correlates of explicit emotion regulation related to traumatic experience

For an overview of studies assessing neural correlates of explicit emotion regulation in trauma-exposed people with and without PTSD, see Table 1.

**Table 1:** Overview of studies assessing neural correlates of explicit emotion regulation or neural plasticity.

S. No.	Study	Sample	Trauma/sample type	Diagnostics	Contrast	Stimuli	Between-group results
1	Bryant et al. (2021)	37 PTSD vs. 24 HC	Assault, childhood abuse, vehicle accidents, police duties	CAPS (DSM-IV)	Reappraisal neg. > watch neg.	IAPS pictures	Whole-brain: ↓ left inferior occipital cortex, left crus I of cerebellar hemisphere, left precentral gyrus, left insula, right middle frontal gyrus, right superior temporal pole, left olfactory cortex, left SMA, right midcingulate area, left thalamus, left middle frontal gyrus, left IFG (pars orbitalis) ROI: ↓ bilateral dlPFC and dmPFC; amygdala n.s.
2	Butler et al. (2019)	18 PTSD vs. 27 TC	Combat	Clinical diagnosis (ICD-10)	Reappraise neg. > feel neg.; suppress neg. > feel neg.^a^	Combat-related images	Whole-brain (reappraise > feel): ↓ rostral ACC/ventromedial PFC (task preparation), ↑ dorsal ACC, occipital cortex (task presentation) Whole-brain (suppress > feel): n.s.
3	Fitzgerald et al. (2017)	28 PTSD vs. 20 TC	Combat	Clinical diagnosis, CAPS (DSM-IV)	Reappraise neg. > maintain neg.	IAPS pictures	NA
4	Keller et al. (2022)	20 PTSD vs. 35 MDD & 34 HC	NA	SCID (DSM-IV)	Reappraise neg. > view neg.	IAPS pictures	Whole-brain: n.s. ROI (PTSD > HC): ↓ right dmPFC and IFG
5	Lee S. W. et al. (2021)	12 TC vs. 15 HC	Childhood maltreatment	-	Regulate (reappraise) neg. > look neg.	Social IAPS pictures	NA
6	Lee K. H. et al. (2021)	40 (TC + PTSD) vs. 41 HC	NA (Refugees)	SCID, CAPS (DSM-IV)	Suppression neg. > look neg.	Socio-affective pictures^b^	Whole-brain: n.s. ROI: all dlPFC, ventrolateral PFC, medial PFC n.s.; only with small volume correction ↑ left lateral PFC
7	Mao et al. (2023)	38 TC vs. 27 HC	Childhood maltreatment	-	Reappraise neg. > view neg.	IAPS pictures	Whole-brain:↓ right orbitofrontal cortex
8	New et al. (2009)	14 PTSD vs. 14 TC & 14 HC	Sexual assault	SCID-I, CAPS (DSM-IV)	Diminish neg. > maintain neg.	IAPS pictures	Whole-brain (PTSD > HC): ↓ bilateral posterior cingulate, left inferior orbital cortex, right superior frontal gyrus, left middle frontal gyrus, medial frontal gyrus, left IFG, left precentral gyrus, left inferior pari lobe; ↑ right IFG, left middle temporal gyrus, left superior temporal gyrus, Rolandic operculum Whole-brain (PTSD > TC): ↓ lingual gyrus, left superior frontal gyrus, left middle frontal gyrus Whole-brain (TC > HC): ↓ right posterior cingulate, bilateral superior frontal gyrus, left middle frontal gyrus, left inferior pari lobe, right caudate; ↑ right middle temporal gyrus, right superior temporal gyrus, right superior occipital gyrus, right inferior occipital gyrus ROI (PTSD > HC): ↓ left lateral PFC and SMA; ACC and intraparietal sulcus n.s.
9	Rabinak et al. (2014)	21 PTSD vs. 21 TC	Combat	SCID-I, CAPS (DSM-IV)	Reappraise neg. > maintain neg.	IAPS pictures	Whole-brain: ↓ left dlPFC, ROI: ↓ dlPFC; n.s. for amygdala, dmPFC, ACC, ventrolateral and ventromedial PFC
10	Schweizer et al. (2016)	23 TC vs. 30 HC	Childhood adversity	Semi-structured interview	Regulate neg. > attend neg.	Film footage	Whole-brain: NA ROI: ↓ amygdala, bilateral middle frontal gyrus, left middle temporal gyrus; n.s. for right IFG, right medial frontal gyrus
11	Sokołowski et al. (2022)	51 (PTSD + TC) vs. 35 HC	Childhood adversity	SCID-I (DSM-IV)	Rumination neg. > abstract	Sentences	Whole-brain: n.s.
12	Steward et al. (2020)	16 PTSD vs. 13 TC & 14 HC	NA	CAPS	No-Think neg. > fixation cross	Faces-IAPS pictures pairs	Whole-brain: ↓ parahippocampal cortex
13	Sullivan et al. (2019)	16 PTSD vs. 19 TC & 13 HC	Combat, child abuse, assault, accident, others’ death	CAPS (DSM-IV)	No-Think > Think, No-Think (forgotten) > Think (remembered)	Faces-IAPS pictures pairs	Whole-brain: NA ROI (PTSD > HC, both contrasts): ↓ right middle frontal gyrus; n.s. for left PFC ROI (TC > HC, both contrasts): ↓ right middle frontal gyrus, n.s. for left PFC ROI (PTSD > TC, both contrasts): n.s.
14	Xiong et al. (2013)	20 PTSD vs. 20 TC	Vehicle accident	SCID-I, CAPS (DSM-IV)	Diminish neg.	IAPS pictures	Whole-brain: ↓ bilateral inferior parietal lobe, right inferior: superior and middle frontal lobes, left putamen, right insula, cuneus
Intervention studies							
1	Fonzo et al. (2017a)	36 PTSD (*trauma-focused exposure*) vs. 30 PTSD Waitlist	Natural disaster, assault, combat injury/ suffering	CAPS, SCID (DSM-IV): pre-post	Reappraise neg. > look neg.	IAPS pictures	No brain activation moderated relationship between treatment arm and symptom change.
2	Fonzo et al. (2017b)						Whole-brain: n.s. ROI: sig. Time × treatment group interaction for left middle frontal gyrus (↑ in exposure group over time)
3	Joshi et al. (2020)	26 PTSD (*exposure + placebo; sertraline; exposure + sertraline*) vs. 24 TC	Combat	CAPS (DSM-IV)—pre-post	Reappraise neg. > maintain neg.	IAPS pictures	ROI (PTSD all treatments > TC, pre-treatment): n.s. ROI (within PTSD all treatments post-pre): n.s. for bilateral amygdala, dmPFC and bilateral dlPFC No comparison of the three treatment groups, no post-fMRI for TC
4	Korgaonkar et al. (2023)	27 PTSD (*trauma-focused exposure*) vs. 21 HC	Assault, childhood abuse, vehicle accidents, police duties	CAPS (DSM-IV) pre-post, MINI	Think (reappraisal) neg. > watch neg.	Traumatic images	ROI: n.s. group × time interaction for left dlPFC
5	Lieberman et al. (2023)	*Neurofeedback:* 14 PTSD vs. 15 HC	NA	CAPS, SCID (DSM-5)	Downregulation neg. > view neg. (PCC)	Trauma-related/ distressing words	Whole-brain: ↓ right dlPFC for training runs; n.s. for transfer run ROI: n.s. for PCC
6	Nicholson et al. (2022)						
7	Nicholson et al. (2017)	*Neurofeedback:* 10 PTSD	NA	CAPS (DSM-5), SCID (DSM-IV)	Downregulation neg. > view neg. (amygdala)	Trauma-related words	No control group
8	Nicholson et al. (2018)	*Neurofeedback:* 14 PTSD	NA	CAPS(DSM-5), SCID (DSM-IV)	Downregulation neg. > view neg. (amygdala)	Trauma-related words	No control group

^aTask preparation and image presentation phase; bKorean Social Affective Visual Stimuli. PTSD, posttraumatic stress disorder; TC, trauma-exposed controls; HC, healthy controls, MDD, Major Depressive Disorder; NA, not available/mentioned; CAPS, Clinically Administered PTSD Scale; SCID, Structured Clinical Interview; DSM, Diagnostic and Statistical Manual of Mental Disorders; MINI, Mini International Neuropsychiatric Interview; neg., negative; PCC, posterior cingulate cortex; IAPS, International Affective Picture System; ROI, regions of interest; SMA, supplementary motor area; dlPFC, dorsolateral prefrontal cortex; dmPFC, dorsomedial prefrontal cortex; IFG, inferior frontal gyrus.^

### 2.1 Reappraisal

**Figure 1:**
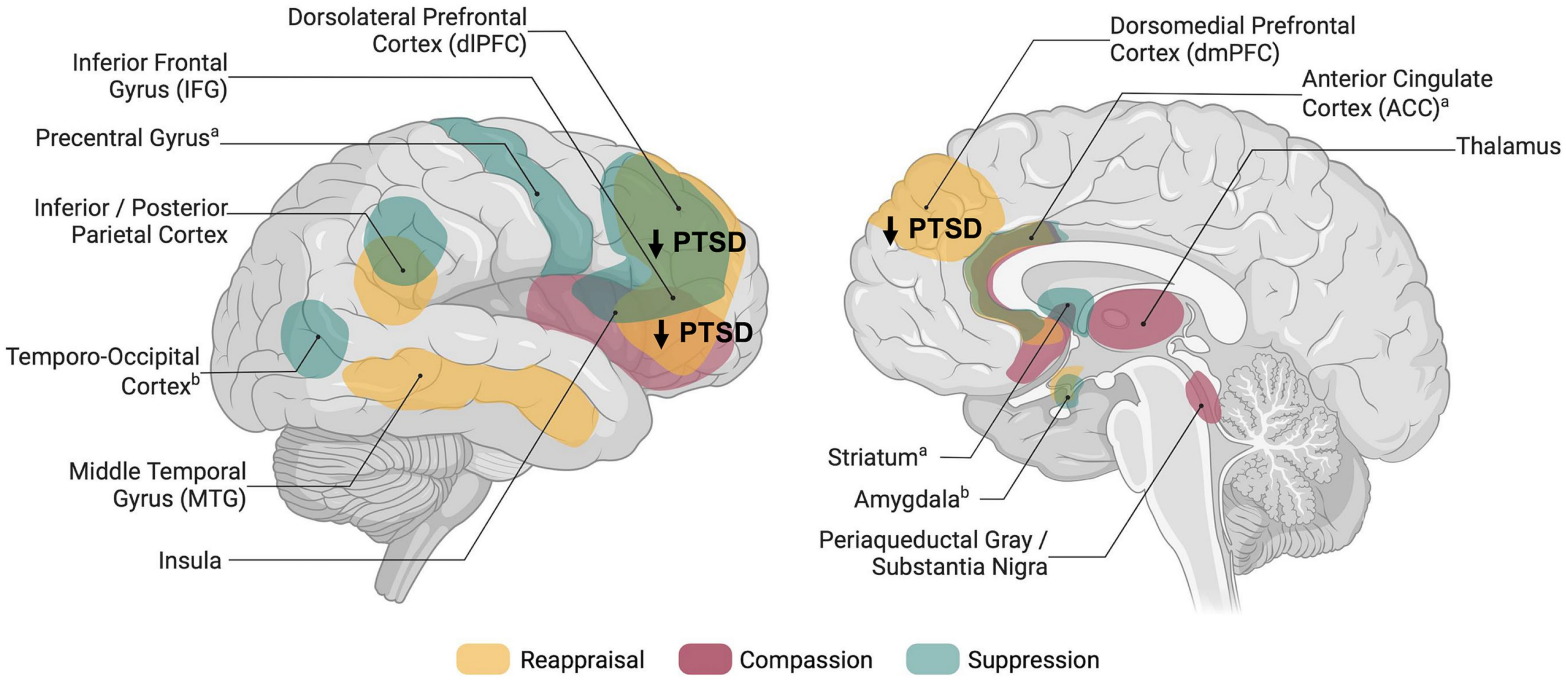
Schematic overview of brain regions associated with explicit emotion regulation in healthy individuals and dysregulation in posttraumatic stress disorder. Networks shown are based on meta-analyses and reviews investigating reappraisal (Buhle et al., 2014), compassion (Kim et al., 2020; Förster and Kanske, 2021), thought/memory suppression (Guo et al., 2018), and expressive suppression (Sikka et al., 2022); ^a^Brain activity related to memory suppression; ^b^Brain activity related to expressive suppression. Figure was created using BioRender.com.

Reappraisal has been defined as an adaptive and antecedent-focused regulatory strategy and describes the process of changing the interpretation of an event that triggers an emotional response (Gross, 1998). In healthy participants, reappraisal engages a network of regions associated with cognitive control, (prefrontal cortex; PFC), conflict monitoring (anterior cingulate cortex; ACC), and semantic processing or perspective taking (middle temporal gyrus; Kanske et al., 2011; Buhle et al., 2014; see Figure 1).

Few studies, even though not explicitly stating that they study reappraisal, instructed participants to “down-regulate” negative emotions (New et al., 2009; Xiong et al., 2013; Schweizer et al., 2016). As the instructions resemble reappraisal, we review these studies together with direct reappraisal instructions. Summarizing the findings in trauma-exposed individuals with PTSD, most studies reported reduced activation of prefrontal regions during reappraisal, suggesting impaired top-down regulatory control during effortful emotion regulation. Specifically, the results showed reduced reappraisal-related activation in key prefrontal areas such as the dorsolateral PFC (New et al., 2009; Rabinak et al., 2014; Bryant et al., 2021), dorsomedial PFC and inferior frontal gyrus (IFG; Bryant et al., 2021; Keller et al., 2022). However, a closer examination reveals that only three studies showed consistent reductions in prefrontal activity in both whole-brain and region-of-interest (ROI) analyses when comparing trauma-exposed individuals with PTSD to healthy controls (New et al., 2009; Bryant et al., 2021) or to trauma-exposed controls without PTSD (New et al., 2009; Rabinak et al., 2014). Other studies, also reported reduced prefrontal activity, but did not observe effects using whole-brain analysis (Keller et al., 2022), or were of lower methodological quality and reported no between-condition contrast (Xiong et al., 2013) or no between-group results (Fitzgerald et al., 2017; Lee S. W. et al., 2021). As such, the results are not specifically attributable to reappraisal or group differences.

Interestingly, one study comparing trauma-exposed controls with and without PTSD distinguished between task instruction and strategy application while measuring brain activity (Butler et al., 2019). In contrast to the expected reduced activity in cognitive control and conflict monitoring regions, they reported *heightened* dorsal ACC activity in PTSD during strategy application. This finding diverges from other studies suggesting that some PTSD patients may exert greater effort during emotion regulation but with potentially reduced efficiency. Nevertheless, they found lower ventromedial PFC activation during the *instruction* phase in PTSD, aligning with theories of reduced regulatory control and highlighting the importance of differentiating between stages of emotion processing.

Notably, reduced brain activity does not necessarily indicate emotion dysregulation, as success is also reflected by reduced negative affect or arousal. For reappraisal, studies showed that people with PTSD reported higher negative ratings than controls (New et al., 2009; Butler et al., 2019). Within-group analyses revealed mixed findings: some reported reduced negative responses following reappraisal (vs. maintain/feel) in PTSD (Rabinak et al., 2014), while others found no differences (Butler et al., 2019). These results complicate interpreting reduced prefrontal engagement as a marker of emotion dysregulation but overall hint a PTSD-specific deficiency in reappraisal. However, when comparing trauma-exposed individuals *without* PTSD to healthy controls, some studies suggest that reduced frontal activation in combination with reduced negative affect (successful downregulation) may be more indicative of efficiency. More specifically, Schweizer et al. (2016) reported that young adults with (vs. without) experiences of early adversity exhibited more successful downregulation in regions such as the amygdala, middle frontal, and temporal areas. Based on this pattern of reduced activity along with effective downregulation of negative emotions, the authors suggested that the early adversity group may have developed a more efficient neural network for emotion regulation, as they were used to manage emotional distress during childhood. In support of this hypothesis, New et al. (2009) showed that trauma-exposed individuals without PTSD exhibited reduced reappraisal-related activity in the left superior and middle frontal gyri compared to healthy controls, while showing no group differences in self-reported affect after reappraisal trials. Similarly, another study reported reduced activity in orbitofrontal regions, but did not report between-group results on reappraisal success (Mao et al., 2023). At a behavioral level, within-group analysis yielded reduced negative affect following reappraisal (vs. maintain), which is also indicative of successful regulation (Rabinak et al., 2014; Butler et al., 2019).

Overall, comparing findings on people with and without PTSD indicate that the reduced prefrontal activity during reappraisal could be a specific effect related to PTSD but not to trauma exposure in general. Correspondingly, some studies also expected changes in the amygdala activation due to the failed prefrontal down-regulation after trauma exposure. However, there were no differences in amygdala activation in trauma-exposed individuals with compared to those without PTSD (Rabinak et al., 2014) or to healthy participants (Bryant et al., 2021) when using whole-brain or ROI analysis.

In summary, studies assessing neural underpinnings of reappraisal related to trauma exposure hint that specifically PTSD appears to be associated with reduced prefrontal engagement, in the dorsolateral PFC. Although there is a growing body of research assessing reappraisal, small sample sizes, lack of reported whole-brain results or between-group contrasts still make it difficult to draw final conlusions considering other brain regions.

### 2.2 Suppression

In contrast to reappraisal, suppression targets the response directly by attempting to inhibit or prevent the full expression of the emotion but seems less effective (Gross, 1998; Gyurak et al., 2011). Similar to reappraisal, suppressing emotional expressions and memories engages prefrontal (e.g., dorsolateral, ventrolateral) and parietal regions (Guo et al., 2018; Sikka et al., 2022; see Figure 1). While expressive suppression has been specifically linked to reduced amygdala and insula activity, suggesting top-down control (Sikka et al., 2022), memory suppression involves striatal activation, indicating inhibitory pathways (Guo et al., 2018). Only few neuroimaging studies instructed participants to suppress negative emotions (Butler et al., 2019; Lee K. H. et al., 2021) or negative memories (Sullivan et al., 2019; Steward et al., 2020). Moreover, one study used instructions to suppress emotions but did not report any related results (Mao et al., 2023). Given the small set of studies, results are much more inconclusive compared to reappraisal trials.

Lee K. H. et al. (2021) reported no differences in prefrontal regions using ROI or corrected whole-brain analysis. However, with small volume correction, refugees (with and without PTSD) compared to healthy controls showed stronger activation in the lateral PFC related to suppression. Hence, refugees may exert more effort to regulate negative emotions, although suppression (vs. the control condition) did not show success on reducing the intensity of negative emotions. Similarly, Butler et al. (2019) reported no differences between combat-exposed individuals with and without PTSD in brain activity at the whole-brain and behavioral level.

Two studies used the Think-/No-Think paradigm, which assesses suppression of aversive memories rather than suppressing emotional responses. Using ROI analysis, Sullivan et al. (2019) found reduced activity in the middle frontal gyrus related to general and successful memory suppression for trauma-exposed people with and without PTSD compared to controls, suggesting a general effect of trauma, not specific to PTSD. In contrast, Steward et al. (2020) did not report similar findings. However, they reported that PTSD patients showed decreased parahippocampal activation during No-Think > Baseline at the whole-brain level compared to healthy controls. Because this contrast does not show brain activity unique to suppressing (vs. thinking about) a memory, it remains unclear whether the effect is suppression-specific or merely due to general attention effects.

In summary, none of these studies reported robust differences between people with and without PTSD and control groups related to emotion or memory suppression. The use of different comparisons, samples (e.g., mixed group with and without PTSD vs. each group separated), and correction methods makes it difficult to aggregate these findings, calling for more research on neural correlates of suppression in trauma-exposed people with and without PTSD compared to healthy controls. Thus, it remains unclear whether potential underlying neural mechanisms of suppression, such as reduced prefrontal activation, are due to the experience of trauma in general or specific to PTSD.

### 2.3 Other emotion regulation strategies

Other explicit emotion regulation strategies have been far less studied, although on a behavioral level various maladaptive regulation strategies have been linked to PTSD, including, rumination, worry, or self-blame (Seligowski et al., 2015; Kaczkurkin et al., 2017). We identified one previous neuroimaging study using a rumination induction task, which showed no differences between individuals with and without adverse childhood experiences, despite differences in functional connectivity were reported (Sokołowski et al., 2022).

Interestingly, one set of adaptive emotion regulation strategies has been neglected altogether in the neuroscientific research of PTSD, that is acceptance and compassion. While acceptance may be described as acknowledgement of the current states without being attached, or judgmental (Messina et al., 2021), compassion is defined as a caring feeling directed towards the suffering of others or to oneself (self-compassion; Neff, 2003; Goetz et al., 2010). When compassion is consciously evoked (e.g., through meditation) to reduce personal distress, it may be conceptualized as explicit emotion regulation (Engen and Singer, 2015). Compassion for others can be a special form of adaptive interpersonal emotion regulation, as it may be used not only to reduce personal distress in social situations, but also to maintain a connection with others (Engen and Singer, 2015). Since the induction of acceptance and compassion is usually associated with mindfulness-based interventions, studies already intersect with intervention research.

We identified one study, directly assessing compassion in people with PTSD though *not* as an explicit regulation strategy, but as direct emotional response towards the suffering of others, reflecting the propensity of compassion. Pino et al. (2016), reported reduced activation in the left anterior insula and left IFG in participants with PTSD compared to trauma-exposed controls during the question of how much empathic concern (compassion) they were feeling in response to pictures of people. This finding supports the idea that training of compassion might be promising target of future research.

## 3 Neural plasticity of explicit emotion regulation following trauma

Training in adaptive explicit emotion regulation is a core component of several interventions for PTSD, utilizing strategies such as reappraisal, but also acceptance and compassion as part of third-wave cognitive-behavioral therapy (Ehlers and Clark, 2000; Hayes and Hofmann, 2017; Karatzias et al., 2023). Although some previous studies indeed examined neural predictors of treatment response (Szeszko and Yehuda, 2019; Manthey et al., 2021), studies including explicit emotion regulation tasks before *and* after treatment to examine neural plasticity are still scarce (see Table 1). Last, the field of real-time fMRI neurofeedback has emerged as potential treatment for PTSD to promote neural plasticity related to regulation of emotion-related brain activation.

### 3.1 Exposure therapy

Fonzo et al. (2017a, 2017b) investigated effects of prolonged exposure therapy on emotion regulation. Using ROI analysis they found a time-by-treatment effect indicating neural plasticity of reappraisal-related activation in the left middle frontal gyrus after prolonged exposure vs. waitlist (Fonzo et al., 2017b). In the same project, they did not find that reappraisal-related brain activity at baseline moderated the effect of treatment on symptom change (Fonzo et al., 2017a). These findings highlight that while exposure is associated with neural plasticity underpinning reappraisal, initial reappraisal-related brain activity seems not to be a marker of who will benefit most from treatment.

Another project assessed reappraisal ability before and after trauma-focused exposure including one session of cognitive reframing (Bryant et al., 2021; Korgaonkar et al., 2023). Contrary to the results of Fonzo et al. (2017b), here reduced dorsolateral PFC activation during reappraisal from pre- to post-treatment was associated with reduced PTSD symptoms after treatment (Korgaonkar et al., 2023). Contrasting the hypothesis of increased prefrontal activation, this finding could be explained by increased efficiency in down-regulating aversive emotions. However, they did not find a reappraisal-related time-by-group interaction, indicating that activity changes were not uniquely driven by the treatment.

In summary, these studies do show neural plasticity related to trauma-focused (exposure) therapy, but the exact mechanism remains unclear, as they observed both increased and decreased prefrontal activation. In contrast, one study combined exposure therapy with placebo or sertraline or applied medical treatment alone, but did not find significant differences between pre- and post-treatment (Joshi et al., 2020).

### 3.2 Mindfulness-based interventions

Mindfulness-based interventions have gained great attention for PTSD treatment (Boyd et al., 2018). Yet, we could not identify studies specifically investigating neural plasticity of compassion or acceptance in trauma-exposed people applying task-based fMRI at pre- *and* post-treatment. We did identify one study reporting increased resting-state connectivity of the posterior cingulate cortex the with dorsolateral PFC and dorsal ACC following mindfulness-based exposure therapy (including self-compassion exercises) in combat veterans with PTSD (King et al., 2016). Although resting-state connectivity is not the focus of our review, these findings provide initial evidence for emotion regulation-related neural plasticity in the context of mindfulness-based interventions in trauma-exposed individuals.

### 3.3 Neurofeedback

Within the last decade, real-time fMRI neurofeedback has shown potential in treating PTSD by promoting direct neuroplasticity. Using neurofeedback, participants are asked to regulate brain activity of a region, for instance, related to emotional experience. Changes in brain activity are visually presented to participants during training runs, followed by transfer runs without visual neurofeedback to assess learning. This form of regulation is—like explicit emotion regulation—a volitional control of the response to an emotional stimulus. When targeting the amygdala, Nicholson et al. (2017, 2018) reported that PTSD patients were able to downregulate amygdala activity in response to trauma-related words. This effect was sustained during transfer run, but did not increase between runs, indicating no learning. However, increased dorsolateral PFC activity between training runs suggested neuroplasticity, though this was not evident when comparing the first training and transfer run (Nicholson et al., 2018). The same research group showed that participants with PTSD and healthy controls were similarly able to decrease brain activity in the posterior cingulate cortex during downregulation vs. viewing of emotional words, without group differences (Nicholson et al., 2022; Lieberman et al., 2023).

## 4 Discussion

Overall, we reviewed neural underpinnings of explicit emotion regulation strategies following trauma and their neural plasticity. Based on the current body of literature, general conclusions on neural underpinnings across explicit emotion regulation strategies cannot be drawn. While reappraisal seems to be associated with a reduced activation in prefrontal brain regions specifically related to PTSD, there is still room for higher quality studies using larger samples sizes and comparing both trauma-exposed people with and without PTSD and healthy controls.

There are general limitations of this review. First, no study had more than 40 participants per group. Given that many fMRI tasks show poor test–retest reliability (Elliott et al., 2020), much larger sample sizes are needed to provide robust estimates. Second, different comparisons lead to different results, as contrasting trauma-exposed individuals with and without PTSD is an option, but also contrasting both groups to healthy or clinical controls. Third, PTSD itself is a heterogeneous disorder including different types and time periods of trauma experience (single vs. prolonged traumatic event, childhood vs. adulthood), which makes aggregation of results more difficult.

Based on this review, we identify the following challenges for future research: research could focus on strategies other than reappraisal, such as compassion, acceptance, rumination, or self-blame. Especially, the question whether there are different neural underpinnings related to different strategies would enhance our understanding of emotion dysregulation following trauma experience. For instance, in healthy participants contrasting compassion directly to reappraisal has revealed activity in the subgenual ACC, mid-insula, and ventral striatum, but not in cognitive control regions, such as the lateral PFC (Engen and Singer, 2015). These distinct neural pathways support the idea that compassion and reappraisal target different aspects of emotion regulation. While reappraisal seems to focus on the antecedent trigger decreasing negative affect, compassion generates positive affect (Engen and Singer, 2015). Notably, explicit emotion regulation is much more than the mere use of a given strategy. The investigation of emotion regulation flexibility, strategy preference, context and goal dependencies could enhance current research and contribute to our general understanding of emotion regulation. Moreover, studies should assess how specific symptoms, symptom clusters, and situational variation may relate to emotion dysregulation on a neural level.

On the intervention research side, there have been promising projects assessing neural correlates of emotion regulation before and after treatment, and others demonstrating the potential of real-time fMRI neurofeedback. However, inconsistent findings related to trauma-focused exposure and lack of learning effects leave room for research. Finally, a general lack of evidence on the neural plasticity of emotion regulation through psychotherapeutic interventions and specifically through mindfulness-based trainings calls for further investigation, as the long-term training of acceptance and compassion could be a promising complement to reappraisal training.
